# Prostate Cancer Stem Cells: Biology and Treatment Implications

**DOI:** 10.3390/ijms241914890

**Published:** 2023-10-04

**Authors:** Ioannis M. Koukourakis, Kalliopi Platoni, Vassilis Kouloulias, Stella Arelaki, Anna Zygogianni

**Affiliations:** 1 Radiation Oncology Unit, 1st Department of Radiology, Aretaieion Hospital, School of Medicine, National and Kapodistrian University of Athens (NKUOA), 11528 Athens, Greece; koukourioannis@gmail.com (I.M.K.); azygogianni@med.uoa.gr (A.Z.); 2 Medical Physics Unit, 2nd Department of Radiology, Attikon University Hospital, School of Medicine, National and Kapodistrian University of Athens (NKUOA), 12462 Athens, Greece; 3 Radiation Oncology Unit, 2nd Department of Radiology, School of Medicine, National and Kapodistrian University of Athens (NKUOA), 12462 Athens, Greece; vkouloul@med.uoa.gr; 4 Translational Functional Cancer Genomics, National Center for Tumor Diseases, German Cancer Research Center, 69120 Heidelberg, Germany; stellaarelaki@gmail.com

**Keywords:** prostate cancer, stem cells, radiotherapy, chemotherapy

## Abstract

Stem cells differentiate into mature organ/tissue-specific cells at a steady pace under normal conditions, but their growth can be accelerated during the process of tissue healing or in the context of certain diseases. It is postulated that the proliferation and growth of carcinomas are sustained by the presence of a vital cellular compartment resembling stem cells residing in normal tissues: ‘stem-like cancer cells’ or cancer stem cells (CSCs). Mutations in prostate stem cells can lead to the formation of prostate cancer. Prostate CSCs (PCSCs) have been identified and partially characterized. These express surface markers include CD44, CD133, integrin α2β1, and pluripotency factors like OCT4, NANOG, and SOX2. Several signaling pathways are also over-activated, including Notch, PTEN/Akt/PI3K, RAS-RAF-MEK-ERK and HH. Moreover, PCSCs appear to induce resistance to radiotherapy and chemotherapy, while their presence has been linked to aggressive cancer behavior and higher relapse rates. The development of treatment policies to target PCSCs in tumors is appealing as radiotherapy and chemotherapy, through cancer cell killing, trigger tumor repopulation via activated stem cells. Thus, blocking this reactive stem cell mobilization may facilitate a positive outcome through cytotoxic treatment.

## 1. Introduction

Normal tissues, with the exception of neurons, are under a continuous renewal state, replacing their aged cells with new cells. Organs sustain a constant number of cells and keep a spatial and functional relationship between the different cellular components within the frame of homeostasis. This genetically predefined process can be disrupted in diseases that either drive cellular and organ degeneration or, on the contrary, promote abnormal proliferation that leads to the appearance of benign or malignant tumors.

This perpetual (throughout the organism’s life) process is based on the conservation of a pre-differentiation cellular compartment endowed since the early steps of organogenesis. Such cells differentiate into mature tissue/organ-specific cells at a steady pace under normal conditions or can accelerate their growth during the healing process or in the context of certain diseases. These clonogenic stem cells can be fully de-differentiated all the way back to the early blastocyst level (embryonal stem cells) or can be characterized by a varying degree of differentiation (adult stem cells) [1].

**Figure 1:**
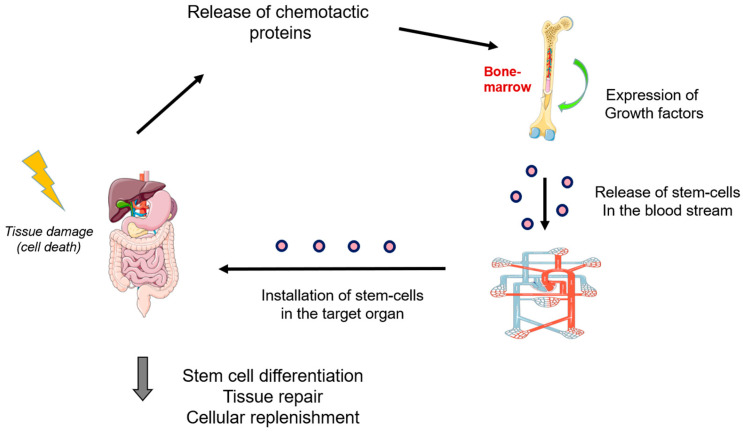
Schematic representation of the activation and installation of bone marrow stem cells in the damaged organs (this figure was partly generated using Servier Medical Art, provided by Servier, licensed under a Creative Commons Attribution 3.0 unported license).

Although they can be found in various organs, the bone marrow is considered the main source of stem cells [2]. Under stressful and damaging conditions, growth factors promote the release of such cells into the bloodstream to reach the target organ. Chemotactic factors released by tissues, like Stromal-Derived Factor-1 (SDF-1), are involved in this process [3]. SDF-1 binds to the CXCR4 receptor on stem cells, inducing the expression of adhesion molecules that facilitate their installation in the damaged organ [4,5,6]. Figure 1 shows a schematic representation of the activation and installation of bone marrow stem cells in the affected tissues.

Carcinomas are not aggregates of abnormal cells. A tumor can be considered a primitive organ as it is composed of the epithelial component that often assumes specific architectural characteristics, like nests or glandular formations, surrounded and supported by the tumor stroma, composed of fibroblasts, vessels and infiltrating inflammatory immune cells. Prostate cancer (PC) is a typical example of adenocarcinoma, an epithelial tumor that in well-differentiated stages resembles normal prostate glands. This normal-like architecture is gradually disorganized as the tumor progresses to a poorly differentiated state. The transition is well described in the Gleason scoring of PC, used by pathologists to describe and predict clinical aggressiveness [7]. It is postulated that in all of these tumor differentiation subtypes, the proliferation and growth of carcinomas are sustained by the presence of a vital cellular compartment resembling normal stem cells: ‘stem-like cancer cells’ or, for simplicity, cancer stem cells (CSCs).

The current review summarizes the theories related to the origin of PC stem cells (PCSCs), their characterization, and the activated molecular pathways. Moreover, it delves into their role in the growth, metastasis and resistance to therapy, and finally, the importance of therapeutic aspects aiming to eradicate this critical for the tumor’s survival cellular compartment.

A literature search was performed using the EMBASE and MEDLINE databases. The following scientific terms were applied to identify experimental studies focusing on PCSCs, translational studies investigating the prognostic and predictive role of the presence of PCSCs in tumor biopsies or surgical specimens, and finally, experimental and clinical studies of therapeutic targeting of PCSCs: “prostate cancer”, “stem cell”, “chemotherapy”, “radiotherapy”, and “androgen deprivation”.

## 2. Cancer Stem Cells (CSCs)

### 2.1. Origins

The first indication of the existence of CSCs was provided from leukemia studies in 1994, where only a specific subpopulation of leukemic cells bearing the CD34+/CD38− phenotype was able to grow after implantation in experimental animal models [8]. The existence of CSCs in solid tumors, like breast and lung cancer or glioblastomas, has been confirmed in subsequent studies [9,10].

**Figure 2:**
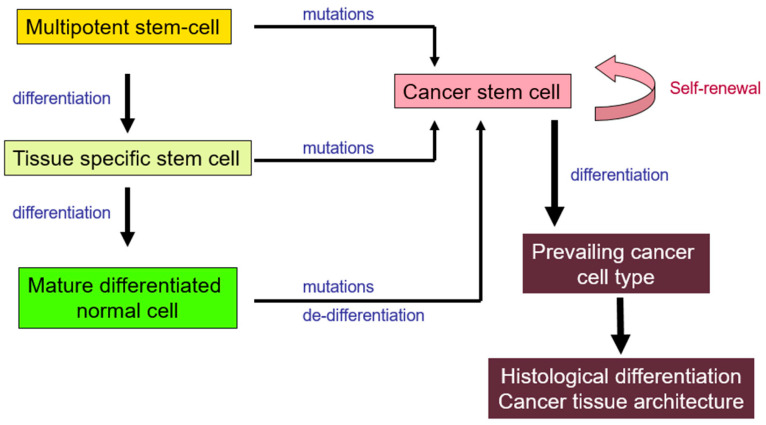
Schematic representation of theoretical models on cancer development and growth from stem cells. Normal multipotent (tissue non-specific) or tissue specific stem cells may undergo mutations that lead to the appearance of cancer stem cells (CSCs). An inverse process could also occur where mutations induced in mature differentiated normal cells promote de-differentiation and transformation to CSCs. CSCs are responsible for the production of cancer cell colonies that will assemble into a growing tissue. CSCs will continue to replenish dying cancer cells and, furthermore, provide the basic phenotype of more differentiated cancer cell offspring that will characterize the histological differentiation.

The origin of these CSCs remains unclear [11,12]. Among the existing theories, CSCs result from spontaneous or carcinogen-induced genetic mutations in normal tissue stem cells. An alternative theory suggests that mutations of differentiated normal cells promote de-differentiation, leading to the emergence of cell clones with stem-like functions. Both theories could be valid. The essence of both theories can be distilled down to tumor growth through continuous proliferation and partial differentiation of stem-like cells. Their function follows a pattern similar to the one of the renewal of normal tissues, albeit lacking the growth restrictions of normal physiology. The progeny cells from these CSCs can either follow a differentiation pathway up to tumor forms with a normal-like appearance or retrogress to fully undifferentiated cell forms, producing a tumor without any recognizable architecture; see Figure 2.

The ability of bone marrow mesenchymal stem cells (BMSCs) to differentiate to mature cells of various organs supports the theory of the origin of CSCs from mutated stem cells [13]. Chronic infections of an organ, e.g., chronic presence of the *Helicobacter pylori* in the gastric mucosa, induce the migration of BMSCs into the stomach [14]. Another example is patients who are recipients of kidney transplants, where mesenchymal stem cells of the donor residing in the transplant can migrate to the skin and produce tumors [15,16]. Overall, BMSCs can be installed in organs and give rise to neoplasias of various histology, including epithelial tumors and sarcomas [17].

The alternative theory of the origin of CSCs from the de-differentiation of mature cancer cells is also valid [18]. Studies have shown that the process of de-differentiation is achievable in laboratory conditions. Breast epithelial cells, for example, can shift to a mesenchymal-type phenotype (epithelial–mesenchymal transition, EMT) resembling breast stem cells [19]. De-differentiation of fibroblasts to multipotent embryonal-line cells is also feasible [20,21]. Moreover, it has been demonstrated that liver and brain tumors can emerge from the de-differentiation of normal liver and glial cells, respectively [22,23]. The biological mechanisms involved in this process are partially known. The Wnt, Hedgehog (HH), and Notch pathways, also involved in carcinogenesis, are important [24,25,26,27].

### 2.2. Tumor Microenvironment

Beyond genetic alterations, the appearance of CSCs demands specific microenvironmental conditions in the organ, which form the ‘stem cell niche.’ This microenvironment is essential for the growth of tumors in distant organs, where circulating cancer cells can be installed and develop metastases. The quality of the stem cell niche depends on the type and function of fibroblasts and endothelial cells and the composition of the extracellular matrix. Moreover, infiltrating immunosuppressive lymphocytes and mononuclear cells may also contribute to the establishment of the unique tumor microenvironment and have discrete interactions with the stem cell niche [28]. This niche provides the signals for CSCs to initiate their growth and differentiation, while simultaneously protecting them from anti-tumor immune assaults [29,30].

An example of a tumοr-friendly niche is the bone marrow, where cancer cells can install early in the course of tumor growth and can survive years after therapy before they give rise to clinically detectable metastases [31]. Hemopoietic cells, fibroblasts, and endothelial cells in the bone marrow support the survival of CSCs residing in it. In addition, mesenchymal stem cells of the bone marrow may play a critical role in the de-differentiation of cancer cells that reach the bone marrow and their transformation to CSCs by inducing stem cell markers like CD133, OCT4 and SOX2 [32,33].

Furthermore, hypoxic stimuli are important drivers of CSCs genesis in the niche. Overexpression of the hypoxia-inducible factors (HIFs) under hypoxia promotes the transcription of various proteins involved in metabolism, angiogenesis, apoptosis inhibition, and invasion. HIFs activate the notch-pathway and the expression of OCT4, which is involved in de-differentiation and stem cell phenotype development [34,35,36,37].

## 3. Stem Cells in Prostate Cancer

### 3.1. Normal Prostate Gland

The glandular epithelium of the normal prostate is composed of four distinct cell types: the secretory (luminal) cells that form the internal layer of the gland, the basal cells that lie directly beneath the secretory cells, the mixed luminal/basal type cells, and finally, the neuroendocrine cells, which are scattered among the secretory cells and constitute the less populated cellular compartment (less than 1% of cells). These prostate glands are surrounded by fibromuscular stroma.

Secretory cells are the most differentiated cells, expressing androgen receptors (ARs), prostate-specific antigen (PSA), and prostatic acid phosphatase (PAP). On the contrary, basal cells are less differentiated, do not express AR, and are considered progenitors of the secretory cells [38]. Basal cells express CD133, CD44 and p63 [39,40,41], and form a heterogeneous cell population that includes stem cells of prostate glands [42]. Trop2 (tumor-associated calcium signal transducer 2)-positive basal cells, for example, possess stem cell properties [43]. Experimental studies have confirmed that basal cells can grow and reproduce the glandular prostate epithelium [44]. On the other hand, the secretory cell layer may also include cells that possess stem cell functions. In PTEN knockout mice, luminal cells overexpressing Sca-1 were identified as drivers of prostate gland hyperplasia [45]. Furthermore, orchiectomized mice have been shown to develop a NKx3-1+ luminal cell population (CARNs, castration-resistant Nkx3-1-expressing cells) that can give rise to prostate glands. Moreover, *Nkx3-1* is essential for the maintenance of stem cell function [46]. Finally, prostate cells expressing integrin α_2_β_1_ can differentiate to basal (PSA^−^CK5/14^+^) and secretory (PSA^+^/AR^+^) prostate cells [47]. Harris et al. recently reported on a PCSC population characterized by CD117 expression [48].

The neuroendocrine cell population is highly differentiated and expresses chromogranin A, synaptophysin, calcitonin and neuron-specific enolase (NSE), but it is deprived of ARs and PSA [49,50]. It has been suggested that p63+ cells give rise to neuroendocrine cells, although it appears that these cells are a common progenitor for all prostate cell types [51]. Whether neuroendocrine cells are formed by specific stem cells or through a trans-differentiation process of luminal cells is unknown [52].

Overall, normal prostate cells expressing CD133, CD117, CD44, and Sca1 can grow in prostate glands once transplanted in immunocompromised mice and, thus, these markers together with integrin α2β1 are considered as hallmarks of normal prostate stem cells [53,54,55,56].

### 3.2. Prostate CSCs (PCSCs) Characterization

PC is often characterized by genomic instability. Fusion of AR-regulated promoters of genes like *TMRPSS2* with the ETS gene family is the most frequent genomic aberration [57]. *TMRPSS2-ERG*, the fusion identified in the majority of cases, has been recorded in PCSCs [58], providing ERG-driven survival advantages, while also being involved in EMT through the AR-ERG-Wnt network [59]. In addition, *PTEN* deletion is a key factor in driving PC development. A total of 20% of PCs and 50% of castration-resistant PCs bear *PTEN* allelic loss or inactivating mutations [60]. Monoallelic loss can lead to prostate intraepithelial neoplasia, but loss of both alleles is required for full malignant transformation [61]. As far as PCSCs are concerned, *PTEN* deletion is considered to support unrestrained growth of PCSCs and tumorigenesis [62].

Mutations in prostate stem cells or differentiated prostate cells can also lead to the formation of PC (directly or following de-differentiation, respectively). Point mutations of the *SPOP* tumor suppressor gene occur in 10% of PC [63]. *SPOP* mutations disrupt SPOP-mediated destruction of Nanog and have been shown to promote PCSC proliferation and tumor progression [64]. *TP53* mutations and amplification of *c-Myc* have further been documented in 7–8% of PCs [65] and are involved in PCSC generation and malignant transformation [66,67]. PC is also characterized by high copy number alterations of genes with transcriptional factor activity that promote stemness of PC cells [68,69]. Finally, aberrant DNA methylation is an epigenetic mechanism frequently noted in PC, responsible for the transformation of normal stem cells to CSCs [70,71].

Moreover, the expression of certain markers, including CD44, integrin α2β1 and CD133, allows PCs to grow in experimental animals [56]. Among these markers, CD133 and CD44 are highly expressed in human PC [72]. It has been suggested, however, that only CD44+/CD24− cells sustain stem cell properties. The CD44+/CD24− subpopulation from the LNCaP cell line shows a distinct gene signature—concerning enzymes (e.g., *ARIH1*, *LDH*), growth factors (e.g., *TGFb1*, *NRG1*), transcription regulators (e.g., *BRF1*, *MYC*, *ETS1*), kinases (e.g., *ERBB4*) and other genes—suggestive of aggressive clinical behavior [73].

Additionally, during androgen deprivation, hormone-dependent LNCaP PC cells can be reprogrammed to stem-like cells expressing CD133, ALDH1A1 and ABCB1A [74]. ALDH1A1 is expressed in secretory and neuroendocrine PCs and is linked to advanced Gleason score, stage and prognosis of patients [75].

Other genes like *SOX2*, *OCT4*, *NANOG*, *SOX9*, and *BMI1* are also overexpressed in PCSCs, as shown in a study of LNCaP and C4-2B PC cell lines exposed to androgen deprivation [76]. Of interest, hypoxia induces these embryonic markers in cancer cells through HIF1α transcriptional activity [77]. Furthermore, EMT (de-differentiation) of PC cells has also been shown to be induced by AR splice variants, like AR-V7, that can be constitutively active in the nuclei independently of androgen regulation [78,79]. Mourkioti et al. recently demonstrated a significant interplay between the GATA2-CDC6 signaling axis and PC senescence; the treatment of androgen-sensitive cells with enzalutamide led to down-regulation of CDC6 through the GATA2 transcription factor and, eventually, to senescence, while enzalutamide-resistant PC cells displayed stable CDC6 levels, EMT activation and a higher invasive potential [80].

### 3.3. Active Biological Pathways in PCSCs

Banerjee et al. have extensively discussed the metabolism and signaling networks of PCSCs [81]. As mentioned above, the *PTEN* tumor suppressor is often de-activated in PC cells. This gene is essential to block the activity of Akt, by down-regulating inositol kinase (PI3K). *PTEN* de-activation promotes Akt-mediated metabolism pathways, proliferation, and apoptosis inhibition [82]. Moreover, the Akt/PI3K pathway is highly activated in half of prostate carcinomas and the majority of metastatic PC cells [83]. Akt is also important in EGFR-driven cell migration and EMT transition in PC [84]. Overall, the PTEN/Akt/PI3K pathway is essential for the maintenance of the PCSC phenotype [82].

Another important pathway is RAS-RAF-MEK-ERK, which is activated in half of all prostate carcinomas, especially in metastatic cases [85]. RAS or RAF mutations that lead to a constitutive activation of the pathway are found in 7–15% of PCs [86]. Binding of the EGFR to its ligands or EGFR mutated state leads to ERK phosphorylation, enhancing the proliferation of PCSCs [87]. In experimental studies, bisphenol-A phosphorylates the ERK kinase and promotes PCSC appearance [88]. In addition, RAS induction in the LNCaP cell line enhances its implantation and growth ability in experimental animals [89]. Moreover, ERK activation suppresses AR expression, leading to the prevalence of de-differentiated PC phenotypes [90].

The signal transducer and activator of transcription (STAT) proteins, like STAT3, also have an important role in the proliferation of PCSCs, as STAT3 inhibitors block the ability of PC cells to grow when implanted in experimental animals [91,92].

Furthermore, the HH signaling pathway controls EMT interactions during embryogenesis, and its activity is often present in PCSCs [93]. Activation of the Notch signaling pathway is also important in sustaining stemness, pluripotency, and self-renewal, while its inhibition during embryogenesis disrupts prostate development. Aberrant Notch signaling controls PCSC proliferation [94,95,96]. The NF-kB pathway is further upregulated in PCSCs [97] and has been linked to resistance to therapy and metastasis [98]. In addition, ABC transporters are overexpressed by PCSCs and contribute to their resistance to chemotherapy [99]. These are also essential to sustain the stem-like phenotype as their inhibition promotes differentiation [100]. Finally, the Wnt/β-catenin pathway is critically involved in PCSC self-renewal and expansion [101].

## 4. Prognostic and Therapeutic Implications

### 4.1. PCSCs and Resistance to Chemotherapy and Radiotherapy (RT)

PC cells expressing the CD44+ stem cell marker are more radioresistant compared to CD44- ones in many PC cell lines. Down-regulation of CD44 delays dsDNA repair and enhances radiosensitivity [102]. Wang et al. demonstrated that irradiation of PC cell lines resulted in a significant increase in CD44+/CD133+ cells that displayed tumorigenic properties in vivo, suggesting that these PCSCs are responsible for the resistance to RT [103]. Guzel et al. studied recurrent prostate tumors after RT and confirmed the prevalence of cancer cells expressing stem cell markers such as OCT4 and SOX2 [104]. In addition, fractionated irradiation of PC cell lines resulted in their enrichment with cells expressing CD133, OCT4 and SOX2 [105]. The percentage of CD133+/CD44+ subpopulation in the DU145 PC cell line increased from 0.1 to 3.5% after exposure to radiation [103]. Increased radioresistance has also been documented for the α2β1-integrin+/CD133+ cancer cells isolated from PC cell lines [106]. Moreover, PCs resistant to docetaxel have high levels of CD44 and CD133 and enhanced invasive and metastatic potential [107].

PC cells expressing CD133/CD44 demand the activity of the Akt/PI3K pathway to sustain this stem-like phenotype. Inhibition of PI3K activity decreases this PCSC population and enhances the sensitivity to docetaxel [108]. In a study by Chang et al., activation of the Akt/PI3K pathway was linked to EMT and resistance to radiation [109], while knock-down of the CD44v6 variant in PC cell lines resulted in suppression of the Akt/PI3K pathway and sensitization of cancer cells to RT and chemotherapy [110].

The Wnt pathway is important in developing resistant cancer cells to RT and chemotherapy [111]. Wnt inhibits apoptosis by activating β-catenin [112]. Wnt/β-catenin signaling induces the transcription of multiple genes related to resistance to chemotherapy, e.g., *MDR-1* and *BIRC5* (survivin) [113]. Cristobal et al. demonstrated that resistance to taxane-chemotherapy regimens for PC cells can also be attributed to this pathway [114]. Furthermore, PC cell lines with active Wnt/β-catenin signaling appear to be less sensitive to anti-androgen therapy [115,116]. Regarding the resistance of PC cells to RT, Cojoc et al. showed that knocking down β-catenin inhibits ALDH activity and restores sensitivity to RT [117].

### 4.2. PCSCs and Prognosis

The identification of stem cells in PC biopsies has been used as a biomarker to predict response to therapy and prognosis. An adverse outcome of patients with PC expressing CD44v5, v3 and v6 isoforms has been reported [118,119]. CD44 overexpression in PC tissue has also been associated with high Gleason score [120]. Moreover, high mRNA levels of prostate stem cell antigen (PSCA) in the cancer tissue of patients treated with prostatectomy have been correlated with a high Gleason score as well as extracapsular and lymphovascular space invasion [121], while an increased presence of PSCA+ PC cells has been linked to high recurrence rates after RT [122]. Over-expression of the stem cell markers CD133 or α6-integrin defined a shorter biochemical relapse-free interval after prostatectomy and high rates of bone metastasis [123,124,125].

The poor prognosis of patients with PC has also been reported in patients with PC expressing high levels of OCT1 and SOX2 [126,127]. Co-expression of SOX9 and HMGB3 that transactivate NANOG was linked to unfavorable outcomes in castration-resistant prostate carcinomas [128]. In addition, high levels of NANOG mRNA in PC tissue have been associated with high preoperative PSA levels, tumor volume, and lymph node metastasis [129]. Other stem cell markers like BMI1 and ALDH1A1 have also been linked to advanced stage and Gleason score [75,130]. Of interest, a negative effect on PC prognosis has been attributed to Gli1 expression, a putative marker of cancer stemness [131].

### 4.3. Targeting PCSCs for Therapy

The development of therapeutic policies to target stem cells in tumors is appealing as RT and chemotherapy, through cancer cell killing, trigger tumor repopulation by activated stem cells. Blocking this reactive stem cell mobilization may become critical for the outcome of cytotoxic treatment.

Signaling pathways involved in stemness are promising targets for the development of such therapies. Blocking the Notch-signaling pathway with monoclonal antibodies (MoAbs) that target Notch ligands and receptors, or γ-secretase inhibitors, suppresses CSC proliferation and sensitizes cancer cells to chemotherapy [132]. Experimental studies have also demonstrated that γ-secretase inhibitors enhance the activity of androgen-deprivation therapy in PC [133,134]. PC cells appeared to be more sensitive to enzalutamide, bicalutamide or abiraterone after treatment with a γ-secretase inhibitor 1 [133]. Of interest, combination of the γ-secretase inhibitor DAPT with enzalutamide led to significant suppression of tumor growth in xenograft PC models when compared to monotherapy with DAPT or enzalutamide [134]. In addition, Notch-signaling blockage restores the sensitivity of PC xenografts to docetaxel [135,136,137]. Qiu et al. demonstrated a more potent inhibition of tumor progression after treatment of PC animal models with the Notch signaling inhibitor DBZ together with docetaxel than the one achieved with either drug alone, hinting at a potential means to overcome docetaxel resistance [135]. Similar effects were reported through the combined use of a γ-secretase inhibitor, PF-03084014, and docetaxel both in vitro and in vivo [136,137]. At the clinical level, a phase I study of RO4929097 γ-secretase inhibitor in PC patients with advanced disease confirmed the good tolerance of the drug. Unfortunately, a phase II trial investigating its combination with bicalutamide was prematurely closed, so no data on clinical efficacy are available [138,139].

In 2023, Wu et al. reported the significant anti-PCSC effects of baicalin through Notch1/NF-kB pathway inhibition in xenograft tumor models that could potentially be exploited for PC treatment [140]. Several clinical trials targeting NF-kB with bortezomib in combination with docetaxel or mitoxantrone have been completed, but the results have either never been reported or are inconclusive (NCT00183937, NCT00193232, NCT00667641).

Several drugs targeting the HH pathway, like vismodegib and sonidegib, have been developed. Clinical trials with these drugs have not confirmed a clear benefit in PC, and their role in combination with RT and chemotherapy has not been examined [141,142,143]. Nevertheless, experimental studies have displayed that HH inhibitors sensitize PC cells to RT [144,145]. Gonissen et al. investigated the effects of the HH inhibitor GANT61 together with irradiation in both PC cell lines and xenografts; in both settings, GANT61 significantly enhanced the cytotoxic properties of RT [145]. Moreover, GANT61 has been reported to downregulate the androgen receptor signaling pathway and sensitize PC cells to enzalutamide [146]. Furthermore, polymeric nanoparticles delivering HH pathway inhibitors have been shown to eradicate CD133+ PCSCs [147].

Wnt-signaling in CSC can also be targeted by small antagonist molecules because of its important role in CSC renewal [148]. More recently, cabazitaxel chemotherapy of PC cells was proven to be more effective when salinomycin and a Wnt inhibitor were used as pretreatment; specifically, this regimen led to reduced cell migration and PCSC content [149].

Surface CSC markers make for an excellent therapeutic target. Anti-CD44 MoAbs have become a target for the development of CSC-targeting therapies [150,151]. In the PC setting, salinomycin nanoparticles attached to CD44 antibodies exerted significant cytotoxic effects to CD44+ PCSCs. Chen et al. further demonstrated that anti-CD44 Campylobacter jejuni genotoxin nanoparticles can potentially enhance the efficacy of RT when treating radioresistant PC through an increase in double-strand breaks and interruption of the cell cycle at the G2/M phase [152,153]. Conjugated anti-CD133 antibodies with cytotoxic drugs or cytotoxic T-cell generated to recognize CD133 are under investigation [154,155]. Tan et al. reported on docetaxel delivering gold nanostars linked to a CD133 antibody that can be used for the treatment of castration-resistant PC [156]. Another interesting therapeutic approach targeting PCSCs is the metronomic administration of chemotherapy agents like topotecan that suppress EMT and de-populate tumors from stem cells [157].

Chimeric antigen receptor (CAR) T-cell therapy has emerged during the past decade as an alternative treatment approach against malignancies that incorporates gene modification of the patient’s T-cells in order to recognize and eradicate cancer cells more effectively. Although certain studies have investigated the role of CAR T-cell therapy for CSCs [158,159], limited data currently exist concerning PCSCs; Deng et al. have reported on the anti-tumor effects of CAR T-cells targeting the PCSC epithelial cell adhesion molecule (EpCAM) both in vitro and in vivo [160].

## 5. Conclusions

PCSCs have been identified and partially characterized. These express surface markers include CD44, CD133, integrin α2β1, and embryonal proteins like OCT4, NANOG, and SOX. Several signaling pathways are activated, including Notch, NF-kB, PTEN/Akt/PI3K, RAS-RAF-MEK-ERK and HH. Documentation of the overexpression of stem cell markers or overactivation of the above signaling pathways has been successfully used to predict the response to therapies and provide prognostic insights. At the therapeutic level, these pathways are under thorough investigation as targets for developing stem cell targeting drugs. Furthermore, new pharmacological technologies like drug delivering nanoparticles conjugated with antibodies against PCSC membrane markers could eventually be utilized to overcome the chemo- and radio-resistance of certain PC subtypes. PCSCs are the critical cellular compartment that replenishes the PC cell population during RT and chemotherapy and are also the most resistant to androgen deprivation therapy. Consequently, the combination of stem-cell-targeting agents is expected to enhance the efficacy of these therapeutic modalities. Tumor recurrence and biochemical relapse after radical therapies like surgery and RT may further be reduced or delayed, should effective anti-PCSC agents become available.

For the time being, the clinical experience with PCSC-targeting agents is limited and does not replicate the encouraging experimental data overall [161]. It is suggested that individual phenotyping of prostate carcinomas to identify the prevalent CSC markers may assist in optimizing clinical trials by recruiting the most susceptible patient subpopulation.
